# Sequenced treatment alternatives to relieve adolescent depression (STAR-AD): a multicentre open-label randomized controlled trial protocol

**DOI:** 10.1186/s12888-023-05221-w

**Published:** 2023-10-27

**Authors:** Yuqian He, Xieyu Gan, Xuemei Li, Ting Wang, Jie Li, Tingting Lei, Yajie Huang, Ruibing Liu, Fei Chen, Teng Teng, Yuxin Xie, Xuan Ouyang, Xinyu Zhou

**Affiliations:** 1 Department of Psychiatry, The First Affiliated Hospital of Chongqing Medical University, 1 Youyi Road, Yuzhong District, Chongqing, China; 2 Department of Neurology, The Second Affiliated Hospital of Chongqing Medical University, Chongqing, China; 3 Department of Psychology, The Second Affiliated Hospital of Chongqing Medical University, Chongqing, China; 4 Department of Psychiatry, The Second Xiangya Hospital, National Clinical Research Center for Mental Disorders, and National Center for Mental Disorders, Central South University, Changsha, China

## Abstract

**Background:**

Adolescent major depressive disorder (MDD) is a prevalent mental health problem with low treatment success rates. Whether fluoxetine or fluoxetine combined with cognitive-behavioural therapy (CBT) is the more effective initial treatment for adolescent MDD remains controversial, and few studies have investigated whether treatment switching or augmentation is preferred when the initial treatment is not working well.

**Methods:**

We developed a multicentre open-label Sequential Multiple Assignment Randomized Trial (SMART) design, consisting of two phases lasting 8 weeks each. In phase 1 (at baseline), patients will be recruited and grouped in fluoxetine group or fluoxetine combined with CBT group by patient self-selection. In phase 2 (after 8 weeks of treatment), the nonresponders will be randomly assigned to six groups, in which participants will switch to sertraline, vortioxetine, or duloxetine or added aripiprazole, olanzapine, or lithium carbonate to fluoxetine. After the full 16 weeks of treatment, we will assess the long-term sustainability of the treatment effects by evaluating participants during their subsequent naturalistic treatment. The primary outcome will be the response rate, determined by the Children’s Depression Rating Scale-Revised (CDRS-R). Secondary outcomes include the change in scores on the Beck Depression Inventory (BDI), the Screen for Child Anxiety-Related Emotional Disorders (SCARED) and the Safe Assessment.

**Discussion:**

The results from this study will aid clinicians in making informed treatment selection decisions for adolescents with MDD.

**Trial registration:**

This protocol was registered at ClinicalTrials.gov with Identifier: NCT05814640.

## Background

Major depressive disorder (MDD) is a prominent factor in disease and disability among adolescents [1], and the prevalence of MDD among adolescents in 2016–2019 was reported to be 4.4% [2]. Considering the burden of this disease, especially in light of the rising rates of suicide and self-harm among adolescent MDD patients [3, 4], early intervention options for this population are urgently needed [5, 6].

Whether fluoxetine or fluoxetine combined with cognitive-behavioural therapy (CBT) is better for treating adolescent MDD remains controversial. The guidelines provided by the National Institute of Clinical Excellence (NICE) in the United Kingdom explicitly recommend prescribing fluoxetine only in conjunction with psychotherapy [7], but the depression practice parameters of the American Academy of Child and Adolescent Psychiatry (AACAP) suggest administration of fluoxetine alone is also a reasonable strategy, and offers no preferred for monotherapy versus combined treatment [6]. Moreover, the Guidelines for Adolescent Depression in Primary Care (GLAD-PC) and Canadian Network for Mood and Anxiety Treatment (CANMAT) recommend combination therapy after patients demonstrate that they are unresponsive to fluoxetine monotherapy [8, 9]. Therefore, more study is urgently required to confirm whether fluoxetine or fluoxetine combined with CBT should be the first choice for treatment.

During acute phase randomized controlled trials (RCTs) with medication administered for 6–8 weeks, it was found that approximately 20–30% of adolescent patients did not respond to fluoxetine or fluoxetine combined with CBT [10]. However, current guidance for adolescent nonresponders, mainly originating from the Treatment of SSRI-Resistant Depression in Adolescents (TORDIA) [11], is supported by little research about switching the initially selected treatment to a different treatment (switch) or adding a nontraditional antidepressant drug (augmentation).

The TORDIA study previously reported that in adolescents with no improvement in depression after one appropriate trial of a selective serotonin reuptake inhibitor (SSRI), there were similar response rates between switching to another SSRI or switching to the norepinephrine reuptake inhibitor (SNRI) venlafaxine. A secondary analysis of the 24-week TORDIA [12] outcomes suggest a potential advantage of early augmentation using an atypical antipsychotic or mood stabilizer, but the sample size used was small. Notably, there are already various evidence-based therapies for adult nonresponders [13, 14], and several guidelines recommend switching or augmentation, providing more options for clinical drug selection [15, 16].

To fill the existing gaps in the knowledge necessary for developing treatment guidelines for adolescent depression, we propose to undertake a Sequential Multiple Assignment Randomized Trial (SMART). This trial design is particularly well-suited for evaluating the effectiveness of treatment regimens in real-world settings and is commonly employed in mental health research. It combines sequential and dynamic treatment methods [17–19]. Our study will consist of two phases. In phase 1, we will compare fluoxetine with the combination therapy (fluoxetine combined with CBT), and in phase 2, adolescent nonresponders will be randomly allocated to switching or augmentation group. Ultimately, we will use patients’ responses and remissions to initial and subsequent treatments, along with demographic data, to derive a prioritized treatment plan for patients undergoing initial treatment and those who are nonresponsive to initial treatment.

**Aims and hypotheses**.

- We aimed to establish adaptive treatment strategies for adolescent MDD patients. We hypothesized that the combination of fluoxetine with CBT yields superior effectiveness compared to fluoxetine alone.
- We aimed to establish suitable treatment approaches for adolescents with MDD who did not respond to their initial antidepressant trial. Our hypothesis suggests that various treatment modalities demonstrate comparable efficacy in alleviating symptoms of depression. However, they may differ in terms of their tolerability, side effects, functional outcomes, and compliance.

## Methods

### Study design

**Fig. 1:**
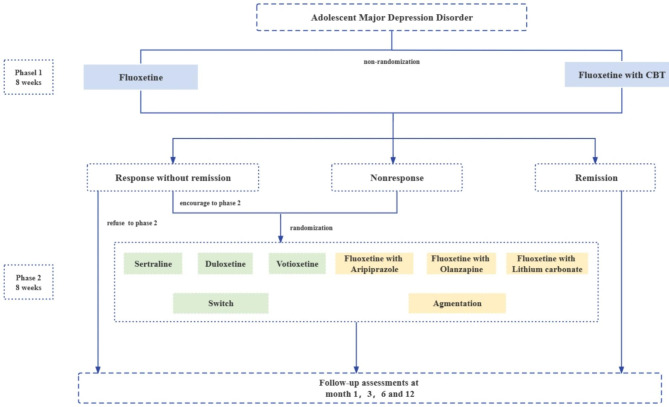
The STAR-AD trial design

Figure 1 depicts the schematic of the trial, which is a multicentre open-label SMART in patients with adolescent MDD. It consists of two treatment phases (phase 1 and phase 2), with each phase spanning 8 weeks, followed by a 12-month naturalistic follow-up phase.

Subjects who complete phase 1 and proceed to phase 2 will include both nonresponsive participants and those who show a response without remission. Nonresponse is determined by a symptom severity reduction of less than 50% as measured by the Children’s Depression Rating Scale-Revised (CDRS-R). Remission, indicating the absence of depressive symptoms, refers to a CDRS-R score of 28 or lower. On the other hand, response without remission is characterized by a reduction of at least 50% from the baseline CDRS-R score, but with a CDRS-R score higher than 28. The clinician determines the transition to phase 2 according to symptom severity, taking into account clinical judgement and considering the total score of the CDRS-R assessment. Participants who do not show a response at the completion of phase 1 may proceed to phase 2, whereas those who achieve remission can proceed to the follow-up phase. Individuals who demonstrate a response but do not achieve remission are also eligible to enter the follow-up phase, although they are strongly advised to move to phase 2 after an adequate dose.

Patients in the follow-up phase will be evaluated over the course of their naturalistic treatment and evaluated for relapse. We established relapse as a situation where there is a single occurrence of a CDRS-R score of 40 or higher, accompanied by a decline in depressive symptoms persisting for a minimum of 2 weeks.

### The rationale of protocol treatments

#### Phase 1

In phase 1, adolescents with MDD will be selected into fluoxetine or fluoxetine combination CBT therapy groups and the choice of treatment will be at the discretion of patient. Some studies have evaluated the effectiveness and safety of these therapies in adolescent MDD [20–22]. Our previous meta-analyses also have shown that for children and adolescents with moderate-to-severe MDD, fluoxetine (either alone or combined with CBT) appears to be the most favourable option for acute treatment [23].

#### Phase 2

In phase 2, sertraline, duloxetine, and vortioxetine are the possible alternative treatments for switching. The reason for selecting sertraline in phase 2 is that a pooled analysis showed that sertraline exhibited greater efficacy compared to a placebo in treating MDD among children and adolescents. Furthermore, the observed side effects were predominantly of mild to moderate intensity, and there were no notable differences in side effects when compared to the placebo [24]. Additionally, according to the NICE and CANMAT 2016 guidelines, sertraline is the recommended second-line treatment. And it is also the most commonly used medicine in public hospitals in China [7, 8, 25]. Duloxetine was chosen based on our previous meta-analysis, which indicated that it has one of the highest likelihoods of being ranked as the most effective [26]. Another meta-analysis supported the consideration of duloxetine as a first-line treatment option [27]. Vortioxetine was selected for its multimodal mechanism of action [28]. Despite a randomized, placebo-controlled study in adolescent MDD treated with vortioxetine showing negative results compared with a placebo [29], there is an open-label study showing that vortioxetine surpass a placebo in terms of effectiveness and is regarded as safe and well-received in adolescents with MDD [30]. In addition, the efficacy of vortioxetine has been well established in adults with MDD in two meta-analyses [31, 32].

For augmentation therapy, aripiprazole, olanzapine, and lithium carbonate were selected. There is a paucity of literature on the augmentation of treatments of adolescent MDD. Thus, we draw considerably on the literature describing adults with MDD who are nonresponders. Aripiprazole was chosen based on its demonstrated efficacy and tolerability as an augmentation agent in adult MDD patients who were nonresponsive [33–35] and has been recommended by several treatment guidelines [16, 36]. Olanzapine was selected based on the findings from two meta-analyses in adults with MDD who were nonresponders, which indicated that augmentation of SSRIs (primarily fluoxetine) with olanzapine results in a greater remission rate in comparison to SSRI alone or a placebo [33, 37]. Lithium carbonate, a mood stabilizer, was chosen for its demonstrated antidepressant efficacy, which has been supported by two meta-analyses of adult MDD patients who were nonresponders [33, 38].

### Participants and recruitment

Participants were recruited at 6 clinical centres in China: The First Affiliated Hospital of Chongqing Medical University, The Second Affiliated Hospital of Chongqing Medical University, Children’s Hospital of Chongqing Medical University, The Southwest Hospital of Army Medical University, The Second Xiangya Hospital of Central South University and Peking University Sixth Hospital. The procedure will be approved and supervised by the committee responsible for overseeing research ethics at each clinical centre. Patients will be recruited from psychiatric outpatient clinics at each centre regardless of their gender, race, or ethnicity mainly. Table 1 outlines the criteria for including and excluding patients with MDD.

**Table 1:** The inclusion and exclusion criteria

Inclusion criteria:
1. Age 13–18
2. As assessed by Kiddie-Schedule for Affective Disorders and Schizophrenia (K-SADS-PL), it meets the DSM-5 criteria for MDD with non-psychotic symptoms
3. Score ≥ 40 on the CDRS-R
4. Participants with suicidal ideation are eligible, as long as clinicians consider outpatient treatment to be safe
5. Sufficient audio-visual level to complete this study
6. Written informed consent was obtained from patients and at least one of their parents
Exclusion criteria:
1. History of bipolar disorder, schizophrenia, autism, eating disorders, primary obsessive compulsive disorder, pervasive developmental disorder, or psychosis not otherwise specified
2. History of serious physical illnesses
3. Substance abuse or dependence
4. Current depressive episode with clear suicidal plans or behaviors
5. Requires inpatient treatment for psychiatric disorders
6. Patients with severe mental illness requiring immediate hospitalization
7. Two or more failed trials of antidepressant drugs: each trial for at least 8 weeks, with the last 4 weeks at full dose (e.g. fluoxetine 40 mg/d, citalopram 40 mg/d, escitalopram 20 mg/d, sertraline 150 mg/d)
8. History of clear-cut intolerability of, or lack of effect with, an adequate trial of at least one protocol treatment option
9. Taking any medicine that contraindicates in combination with or interferes with the efficacy of the treatment
10. Taking or administering antidepressants within 5 half-lives
11. Received modified electroconvulsive therapy within 12 months
12. Female patients with pregnancy

### Intervention

#### Pharmacological treatments

In phase 1, adolescents with MDD will be administered flexibly dosed fluoxetine (10–60 mg/day) for a duration of 8 weeks. The treatment will commence with a daily dose of 10 mg, with doses increasing to 40 mg/d at week 4 for those who have not achieved a ≥ 50% reduction in CDRS-R score. The decision to further increase the fluoxetine dose (maximum dose should not exceed 60 mg/d) will be at the discretion of the physician and depend on the patient’s condition.

In phase 2, some adolescent MDD patients will switch to a flexible dosage of either sertraline (25–200 mg/d), duloxetine (10–60 mg/d), or votioxetine (5–20 mg/d) for a period of 8 weeks. For patients who do not experience a reduction of at least 50% in CDRS-R score by week 4, the drug dosage will be adjusted to 150 mg/d for patients taking sertraline, or 40 mg/d for patients taking duloxetine, or 15 mg/d for patients taking vortioxetine. For other patients, augmentation will involve the flexible addition of either aripiprazole (2.5–15 mg/d), olanzapine (1.25–10 mg/d), or lithium carbonate (125–500 mg/d) to fluoxetine (40–60 mg/d). The decision to further increase the dose (not exceeding the maximum dose) will be at the discretion of the physician and depend on the patient’s condition.

This standardized protocol mirrors routine clinical practice and will promote uniformity across all clinical sites. The adherence to medication will be tracked through pill counts conducted at every visit, and the treatment will be open-label. In cases where patients are affected by severe anxiety or insomnia, appropriate sedative and hypnotic drugs (Table 2) may be administered; clinicians will be required to document the reasoning behind such a prescription. Notably, discontinuing or altering the antipsychotic medication will be considered quitting the research.

**Table 2:** The recommended dosing for sedative and hypnotic drugs

Medications	Recommended dosing
Alprazolam	0.2-0.4 mg/day
Clonazepam	1-2 mg/day
Lorazepam	0.5-1 mg/day
Zaleplon	5-10 mg/day
Zolpidem	5-10 mg/day
Zopiclone	3.75-7.5 mg/day

If patients with severe anxiety or severe insomnia are combined, symptomatic drugs can be given as appropriate. Frequency of use: no more than 2 weeks, recording time and reason

#### Psychotherapy

Adolescent MDD patients who enter the combined treatment group in the first phase will receive CBT and fluoxetine. The CBT manual, developed and revised by experienced psychologists, consists of 7 core modules, including psychoeducation, behavioural activation, mood monitoring and management, improving social skills, cognitive restructuring, negotiation and problem solving, and maintaining gains. The treatment process will take place in two formats; the main format will be adolescent and parent-adolescent groups meeting for a total of 11 sessions lasting 90–120 min each over 8 weeks. (Table 3). Our psychotherapists will receive regular supervision to ensure rigorous psychotherapy processes, and all treatment sessions will be videotaped.

**Table 3:** CBT treatment plan

Module title	participant	Module content
Psychoeducation	A, P	Psychoeducation on depression, dictate the goals for therapy and clarify the group-therapy setting
Behavioral activation I	A	Teaches how to identify simple, realistic and achievable pleasant activity to implement
Mood Monitoring and Management	A	Teaches how to do mood monitoring and relaxation techniques
Social Skills I	A	Teaches how to join a conversation, and when to start conversations
Social Skills II	A	Teaches and practice a variety of techniques such as active listening, receive and express compliments etc.
Behavioral activation II	A	Teaches cognitive behavioral therapy for insomnia (CBT-I)
Cognitive restructuring I	A	Teaches how to identify negative and irrational thoughts, identify and adjust parts of the cognitive distortions
Cognitive restructuring II	A	Teaches how to identify and adjust the other part of cognitive distortions and form one or more realistic thoughts to counter these
Negotiation and Problem-solvingI	A	Teaches how to identify problems, find different solutions, and choose a compromise plans
Negotiation and Problem-solvingII	A, P	Teaches family members reasonable management patterns, reduce blame and increase trust
Maintaining Gains	A, P	Learn to self-monitor long-term emotions and prepare as a family before a stressor arrives.

A = Adolescent; P = Parents

### Study outcomes

#### Baseline

During the screening phase, each patient will undergo a comprehensive psychiatric consultation to verify the diagnosis of MDD based on the Diagnostic and Statistical Manual of Mental Disorders, Fifth Edition (DSM-5) criteria. Additionally, their medical history (including present and previous therapies) will be collected, and for females, their menstrual/reproductive history will also be documented. A medical assessment will be conducted, including height and weight measurement, laboratory tests, physical examination, and 12-lead ECG, as well as collection of basic information about demographics, race, education, social relationships, children trauma, domestic violence, school bullying, depressive symptoms, rumination, and suicidal self-injury will be required for participants. Participants’ magnetic resonance imaging (MRI) contraindications will also be noted.

### Clinical platform

Adolescent MDD patients will attend 8 follow-up visits to perform clinical examinations and assessments according to the schedule (Table 4). The examinations and assessments were selected considering their theoretical and clinical usefulness while minimizing the burden on respondents. Raters underwent training, and the reliability between different raters has been established through the use of recorded interviews.

**Table 4:** Schedule of events

item		Screening	Treatment						Follow-up			
			Level 1				Level 2					
Instruments		V0	V1	V2	V3	V4^a^	V5	V6^a^	V7	V8	V9	V10
Informed consent		√										
Clinical diagnosis		√										
Physical examination		√			√	√	√	√				
Demographic information			√									
Record drug combination			√									
Electrocardiogram examination (ECG)		√										
MRI scan (if necessary)		√										
Laboratory examination^b^		√				√		√				
Measures and assessment												
CDRS-R^c^		√	√	√	√	√	√	√	√	√	√	√
BDI^d^			√		√	√	√	√	√	√	√	√
SCARED^e^			√		√	√	√	√	√	√	√	√
C-SSRS^f^		√	√		√	√	√	√	√	√	√	√
PSQI^g^			√			√		√	√	√	√	√
PedsQL^h^			√			√		√	√	√	√	√
HCL-32^i^			√		√	√	√	√	√	√	√	√
RSS^j^			√			√						
CGI-S^k^			√		√	√	√	√				
CGI-I^k^					√	√	√	√				
CTQ^l^			√									
OB/VQ^m^			√									
MMAS-8^n^					√	√	√	√	√	√	√	√
AEs				√	√	√	√	√	√	√	√	√
SAEs				√	√	√	√	√	√	√	√	√

^a^ V4 and V6 refer to the end of phase 1 and 2, respectively; phase 1 and 2 responders are followed for 12 months at the end of the 16-month treatment period; those who do not respond in phase 1 are followed for the next phase with a new treatment, monthly follow-up. Regardless of response at the end of phase 2, they will be followed for 12 months at the natural follow-up

^b^ Laboratory tests include routine blood, urine and stool tests, liver and kidney function tests, and thyroid function tests

^c^ CDRS = Children’s Depression Rating Scale

^d^ BDI = Beck Depression Inventory

^e^ SCARED = Screen for Child Anxiety-Related Emotional Disorders

^f^ C-SSRS = Columbia Suicide Severity Rating Scale

^g^ PSQI = Pittsburgh Sleep Quality Index

^h^ PedsQL4.0 = Pediatric Quality of Life Inventory 4.0 generic core scales

^i^ HCL-32 = Hypomania Symptom Checklist-32

^j^ RSS = Ruminative Responses Scale

^k^ CGI-S = Clinical Global Impressions-Severity;CGI-I = Clinical Global Impressions-Improvement

^l^ CTQ = Childhood Trauma Questionnaire-Short Form

^m^ OB/VQ = Olweus Bully/Victim Questionnaire

^n^ MMAS-8 = Morisky Medication Adherence Questionnaire

### Primary outcomes

#### Children’s Depression Rating Scale (CDRS-R)

The primary outcomes are the treatment remission rate and response rate. The CDRS-R is a scale consisting of 17 items that are evaluated by clinicians to assess the intensity of depressive symptoms in adolescents, with items scored on scales of 1 to 5 or 1 to 7, resulting in a possible total score range of 17 to 113 [39].

### Secondary outcomes

#### The Beck Depression Inventory (BDI)

The BDI is a self-report rating scale containing 21 items. Based on the BDI scores, depression can be classified into six levels: normal (1–10), mild mood disorder (11–16), borderline clinical depression (17–20), moderate depression (21–30), severe depression (31–40), and extreme depression (over 40) [40].

### The screen for child anxiety-related emotional Disorders (SCARED)

The SCARED is employed to evaluate child anxiety symptoms. Each question is rated from 0 to 2, with 0 denoting “not true or hardly ever true,” 1 denoting “somewhat true or sometimes true,” and 2 denoting “extremely true or often true” [41, 42]. The maximum total score is 63. The recommended total score cut-off of 25 points in Chinese children was used to group patients with and without anxiety symptoms, with higher scores indicating a greater number of anxiety symptoms.

### Columbia suicide severity rating scale (C-SSRS)

The measurement of suicidal ideation involves the use of the C-SSRS, a semi-structured clinician-rated interview specifically designed to evaluate the severity of suicidal behaviour and ideation among individuals aged 11 years and older in various settings, including research, clinical, and community environments [43, 44].

### Hypomania Symptom Checklist-32 (HCL-32)

The HCL-32 is an excellent tool to screen for past episodes of hypomania developed by Jules Angst [45, 46]. It consists of 32 items describing symptoms of hypomania which require a yes/no answer based on whether the participant experienced that symptom. The final score is derived by summing the affirmative answers, and patients with a total score of 13 or higher are identified as having probable bipolar disorder (BD).

### Pittsburgh Sleep Quality Index (PSQI)

The PSQI is self-administered questionnaire with 19 items that evaluate sleep quality and disturbances more than one month. It includes seven “component” scores: daytime dysfunction, sleep latency, use of sleeping medication, subjective sleep quality, sleep disturbances, sleep duration, and habitual sleep efficiency [47–49]. Participants rated each item on a scale from 0 to 3, with 0 indicating that the experience did not occur in the past month and 3 indicating that it occurred three or more times a week. The PSQI total score spans from 0 to 21, and if the global score exceeds 5, it suggests subpar sleep quality.

### The Pediatric Quality of Life Inventory 4.0 generic core scales (PedsQL4.0)

The PedsQL4.0 is a standard questionnaire used to evaluate the quality of life over the previous week in population aged 8 to 18 [50]. There are 23 items in total, and items are divided into four categories: physical functioning, emotional functioning, school functioning, and social functioning. The total score of the PedsQL is determined by averaging all the items across the entire questionnaire, with a range from 0 to 100. Higher scores signify a higher quality of life.

#### Ruminative responses Scale (RSS)

The RRS comprises 22 items that assess three aspects of rumination (brooding, symptom rumination, and reflective pondering); each item is rated on a scale from 1 to 4 [51, 52]. The total score on the RRS spans from 22 to 88, with higher scores indicating greater intensity of rumination.

### Clinical global impressions-severity (CGI-S)

The CGI-S evaluates the degree of global functional impairment, including but not restricted to issues with social interaction and internalizing and externalizing issues [53]. A treating practitioner determines the CGI-S rating on a 7-point scale, with 1 being normal and 7 being among the most severe.

### Clinical global impressions-improvement (CGI-I)

The improvement in overall functioning over the past week since the start of treatment will be measured using the CGI-I [53]. A treating practitioner will determine the score based on a 7-point scale, with 1 denoting a very significant improvement, 7 denoting a very significant deterioration, and 4 denoting no change. Clinical judgement will be guided by all available information.

### Childhood trauma questionnaire-short form (CTQ)

The CTQ questionnaire comprises 28 items that assess different forms of childhood trauma, including sexual, physical, and emotional abuse, as well as emotional and physical neglect. The questionnaire is used to evaluate childhood trauma, where respondents rate each item on a five-point Likert scale that ranges from “Never” (assigned a value of 1) to “Always” (assigned a value of 5) [54]. The overall score was calculated by adding up the scores of all the items, and higher scores indicated a greater frequency and severity of child maltreatment experiences prior to the age of 16.

### Olweus Bully/Victim questionnaire (OB/VQ)

The OBVQ is a self-report questionnaire with 42 items, we selected 7 of the questions from the school bullying section for the experiment in order to perform a simple survey of the school bullying situation of adolescent patients with depression [55]. All answers were scored as a Likert 5-point scale: several times a week, once a week, two or three times per month, once or twice, and never happened.

### Adherence

The Morisky Medication Adherence Questionnaire-8 (MMA-8) is a widely utilized and dependable measure for assessing medication adherence. It comprises eight questions, each targeting a distinct aspect of adherence behaviour, with a total score range of 0 to 8 [56]. The first seven items are answered with a “yes” or “no,“ while the final item offers five response options. Adherence levels are classified as low, moderate, or high based on MMAS-8 scores of < 6, 6 to 8, and 8, respectively [56–58]. And pill count also will be used to enhance validity of data (Adherence was considered to be poor with a tablet count of less than 80% or more than 120%).

The MMAS-8 Scale, content, name, and trademarks are protected by US copyright and trademark laws. Permission for use of the scale and its coding is required. A license agreement is available from MMAR, LLC.,www.moriskyscale.com.

### Safety assessment

Safety will be assessed with all-cause discontinuation, which encompasses both side effects and efficacy. All-cause discontinuation refers to discontinuation for any reason, such as insufficient effectiveness, intolerable adverse effects, or poor adherence. Adverse events (AE) and Significant adverse events (SAE) are defined as in previous studies, such as the Treatment for Adolescents With Depression Study (TAD) [10]. Adverse events will be collected after the subject has provided consent and enrolled in the study. If a subject experiences an adverse event after the informed consent document is signed (entry) but the subject has not started to receive study intervention, the event will be reported as not related to study drug. All adverse events occurring after entry into the study and until finish the study will be recorded. An AE that meets the criteria for SAE between study enrollment and hospital discharge will be arbitration by the STAR-AD Clinical Events Safety Committee which constitute with three independent clinical event reviewers. Side-effect discontinuations will also be recorded.

### Molecular platform

Blood, urine, saliva, and faecal samples will be obtained from all participants during the initial assessment, as well as at weeks 8 and 16. Samples will be processed and stored at the Chongqing Medical University. The laboratory adheres to Good Clinical Laboratory Practice and Good Manufacturing Practices, and follows standardized Standard Operating Procedures (SOPs) and protocols for receiving and processing samples. The samples will be frozen at -80 °C for later testing.

### Neuroimaging platform

Adolescent MDD patients will undergo 3 or 4 imaging sessions using a 3T MRI scanner: (1) before treatment initiation (baseline), (2) following 4 weeks of treatment, (3) following 8 weeks of treatment, and (4) following 4 months of treatment for responders. At each session, data from resting-state functional MRI, structural MRI, and diffusion tensor imaging will be collected. Neuroimaging will be performed to discover potential predictors of response and nonresponse in adolescent MDD and investigate alterations in brain characteristics throughout phase 1 and 2 of the treatment.

### Sample size

According to the preliminary pilot study, we hypothesized that the response rates of fluoxetine and combination therapy in stage 1 were 40% and 60%, respectively. The significance level (α) was set at 0.025 (one-tailed), the test power (1-β) was set to 0.8, and the distribution ratio of fluoxetine and combination therapy in phase 1 was established as 1:1 according the preliminary pilot study. Considering a 20% dropout rate, the sample size must be greater than 238.

However, in order to achieve statistical significance, phase 2 of clinical trials necessitates a minimum sample size of at least 30 patients in each group [59]. Considering a 20% dropout rate, the sample size going into phase 2 cannot be less than 216. Therefore, combining the sample size from both stages and accounting for the response rate in the first phase, our sample size target for this trial is 520 patients.

#### Randomization

After week 8 of the Phase 1 intervention, nonresponding patients and those with a response without remission will be randomly assigned by central allocation. The randomization sequence will be created using StataPM.14 software and stratified by the centre, ensuring these patients will be ramdomly equally assigned to one of six treatment groups. To simulate a clinical environment reflecting real-world conditions, the medications are administered in an open-label manner with flexible dosing. The patient, psychiatrist, and outcome assessor are aware of the group allocation, whereas the data analyst will remain blinded to this information. The clinicians providing the intervention will not conduct the evaluation. All investigators, staff, and participants will maintain strict confidentiality regarding the outcome measures and trial results.

#### Data collection and management

We will use Yiducloud (https://www.yiducloud.com.cn/), a secure and extensively utilized web-based research application, for data collection and entry. The application offers a user-friendly interface for accurate data entry, allows tracking of data manipulation through audit trails, and provides automated export functions for convenient data downloads to Excel. Our database will require complete responses to all interview questions and survey, and will include tools to bring in data from external sources.

We will implement a robust data management strategy to ensure the reliability and availability of the study data. All participant information will be securely stored in a Yiducloud database and managed for efficient updating, tracking, and exporting of data in various formats. We will implement procedures to ensure compliance with local institutional review boards and manage data requests through a data use agreement.

Data entry will be conducted by a trained officer and each data entry is then carefully double-checked by another research team member. Data completeness will be reviewed monthly. Reasons for missing data will be documented in real time during data collection and entry, and a second reviewer will review and verify 10% of the data for quality control. This plan will ensure the reliability of data used for analysis and dissemination while ensuring ethical and regulatory compliance.

### Statistical analysis

The principle of intention to treat, safety set, per-protocol set, full analysis set will be incorporated into the analysis. All statistical tests will be performed as two-sided tests, and *P* value below 0.05 will be deemed as indicating statistical significance. Quantitative indicators will be described by their mean and standard deviation. Classification indicators will be described by the number of cases and percentages in each category. To evaluate the impact of treatments on primary and secondary effectiveness endpoints, measured at baseline, 8 weeks, and 16 weeks, analysis of covariance will be utilized for quantitative data, considering the baseline measurement as a covariate. For qualitative data, a chi-squared test adjusted for central effects will be employed to compare groups.

## Discussion

This protocol describes the reasoning, purpose, and design of the trial. The objective of this trial is to examine the effectiveness and safety of different treatment regimens for depression in adolescents who are initially treated successfully or are nonresponsive. The findings from this trial will offer valuable evidence for clinicians in their choice of monotherapy or combination therapy for adolescent MDD patients. It will also offer evidence for identifying patients who do not respond to initial treatment and contribute to the understanding of the effectiveness and safety of antidepressants with various mechanisms and booster agents. The study employs a combination of dynamic therapy and sequential therapy, making it well-suited for evaluating the effectiveness of treatment options in real-world clinical settings. At the same time, we will also include a rubric for specific symptoms associated with depression, such as anxiety symptoms, suicide risk, sleep status, and quality of survival, and will also analyse the short-term effects of treatment over 6 months and the long-term effects over 12 months, including by evaluating relapse rates. Therefore, we hope that this study can provide guidance in developing optimal treatment protocol for adolescents with MDD.

## Data Availability

Data sharing is not applicable to this article as no datasets were generated or analysed during the current study.

## Abbreviations

STAR-AD: Sequenced Treatment Alternatives to Relieve Adolescent Depression (STAR-AD)
MDD: major depressive disorder
CBT: cognitive-behavioural therapy
SMART: Sequential Multiple Assignment Randomized Trial
CDRS-R: the Children’s Depression Rating Scale-Revised
BDI: the Beck Depression Inventory
SCARED: the Screen for Child Anxiety-Related Emotional Disorders
NICE: the National Institute of Clinical Excellence
AACAP: the American Academy of Child and Adolescent Psychiatry
GLAD-PC: the Guidelines for Adolescent Depression in Primary Care
CANMAT: Canadian Network for Mood and Anxiety Treatment
RCT: randomized controlled trial
TORDIA: the Treatment of SSRI-Resistant Depression in Adolescents
SSRI: selective serotonin reuptake inhibitor
SNRI: norepinephrine reuptake inhibitor
DSM-5: the Diagnostic and Statistical Manual of Mental Disorders, Fifth Edition
MRI: magnetic resonance imaging
C-SSRS: Columbia Suicide Severity Rating Scale
HCL-32: Hypomania Symptom Checklist-32
PSQI: Pittsburgh Sleep Quality Index
PedsQL4.0: The Pediatric Quality of Life Inventory 4.0 generic core scales
RSS: Ruminative Responses Scale
CGI-S: Clinical Global Impressions-Severity
CGI-I: Clinical Global Impressions-Improvement
CTQ: Childhood Trauma Questionnaire-Short Form
OB/VQ: Olweus Bully/Victim Questionnaire
MMA-8: The Morisky Medication Adherence Questionnaire-8
AE: Adverse events
SAE: significant adverse events
SOPs: Standard Operating Procedures
